# Circulating lymphocyte number has a positive association with tumor response in neoadjuvant chemoradiotherapy for advanced rectal cancer

**DOI:** 10.1186/1748-717X-5-47

**Published:** 2010-06-03

**Authors:** Joji Kitayama, Koji Yasuda, Kazushige Kawai, Eiji Sunami, Hirokazu Nagawa

**Affiliations:** 1 Department of Surgery, Division of Surgical Oncology University of Tokyo, Japan

## Abstract

Although neoadjuvant chemoradiotherapy (CRT) is the standard treatment for advanced rectal cancer (RC), markers to predict the treatment response have not been fully established. In 73 patients with advanced RC who underwent CRT in a neoadjuvant setting, we retrospectively examined the associations between the clinical effects of CRT and blood cell counts before and after CRT. Clinical or pathological complete response (CR) was observed in 10 (14%) cases. The CR rate correlated significantly with the size and the circumferential extent of the tumor. Hemoglobin level, white blood cell (WBC) count and platelet count before CRT did not show a significant difference between CR and non-CR cases. Interestingly, however, lymphocyte ratio in WBC was significantly higher (p = 0.020), while neutrophil ratio tended to be lower (p = 0.099), in CR cases, which was shown to be an independent association by multivariate analysis. When all the blood data obtained in the entire treatment period were evaluated, circulating lymphocyte count was most markedly decreased in the CRT period and gradually recovered by the time of surgery, while the numbers of neutrophils and monocytes were comparatively stable. Moreover, the lymphocyte percentage in samples obtained from CR patients was maintained at a relatively higher level than that from non-CR patients. Since tumor shrinkage is known to be dependent not only on the characteristics of tumor cells but also on various host conditions, our data raise the possibility that a lymphocyte-mediated immune reaction may have a positive role in achieving complete eradication of tumor cells. Maintenance of circulating lymphocyte number may improve the response to CRT in rectal cancer.

## Findings

Preoperative chemoradiotherapy (CRT) is currently used worldwide as the initial treatment for advanced RC, since it can produce down-staging in approximately half of patients with locally advanced rectal cancer RC, resulting in a lower rate of postoperative local recurrence and a higher rate of sphincter-preserving surgery as well as longer survival [1-3]. However, in unresponsive cases, it may have disadvantages such as delaying surgery or immune suppression. Although many clinical factors [4,5], radiologic findings [6,7] and molecular markers [7-10] have been suggested to be related to the therapeutic response, the clinical usefulness of these markers remains controversial, and thus, identifying factors that can predict the efficacy of neoadjuvant CRT is essential for decision-making in the management of patients with RC.

In this study, we retrospectively examined circulating blood cells before and after CRT and assessed the possible relationship between these laboratory values and tumor response to CRT, with the approval of the Ethics Committee of the University of Tokyo. Seventy-three patients with rectal adenocarcinoma newly diagnosed between November 2004 and August 2009 received CRT at Tokyo University Hospital. All the patients received a total dose of 50.4Gy radiation and concomitant 5-FU-based chemotherapy. Peripheral blood data were investigated from the medical records of these patients. Pre-CRT blood data were obtained from samples collected 0-53 days before the start of CRT, and all the blood data during the period from the start of CRT to surgery were also examined in each patient. Of the 73 patients, 69 underwent total mesorectal excision in the Department of Surgical Oncology. In 7 cases, no tumor cells were detected at either the primary site or in regional lymph nodes on pathological examination, confirming pathological complete response (pCR). Three other patients showed a clinical CR (cCR) after CRT, with no detectable cancer cells on multiple biopsy specimens, and were thus followed without surgery and showed no evidence of recurrence for more than 12 months, and were also included in the CR group. The clinical and pathological data of the 10 CR and other 63 non-CR cases are shown in Table 1. Patients with tumors with circumferential size more than 4.0 cm determined by computed tomography (CT) showed a significantly lower CR rate than those with tumors less than 4.0 cm (p < 0.05). Also, tumors with a circumferential extent of more than 60% determined by colonoscopy were relatively resistant (p < 0.05). However, none of the other factors, including chemotherapeutic regimen, was significantly associated with the CR rate.

**Table 1:** Correlation between clinical and pathological factors before CRT and pathologica Response in rectal cancer patientsl

	Non-CR (63)	CR (10)	p value
			
Age (years)	63.4 ± 9.9	65.4 ± 11.2	0.455
Sex			
Male	39	6	0.908
Female	24	4	
T stage			
2	12	1	0.901
3	45	8	
4	6	1	
N stage			
0	49	9	0.487
1	14	1	
Clinical stage			
≤2	48	9	0.327
≥3	15	1	
Histology			
Differentiated	61	9	0.313
Undifferentiated	2	1	
Size			
≤40 mm	29	8	0.046*
>40 mm	34	2	
Circumferential extent			
≤60%	27	8	0.029*
>60%	36	2	
Distance from anal verge			
>5 cm	21	5	0.307
≤5 cm	42	5	
Chemo regimen			
UFT+LV	55	6	0.148
5Fu	4	2	
S1	4	2	
CEA			
>5.0 ng/ml	35	3	0.120
≤5.0 ng/ml	27	7	

The size of the tumor was defined as the largest diameter determined by CT, and circumferential extent and distance from the anal verge were determined by colonoscopy

performed before CRT

*: p < 0.05

**Figure 1:**
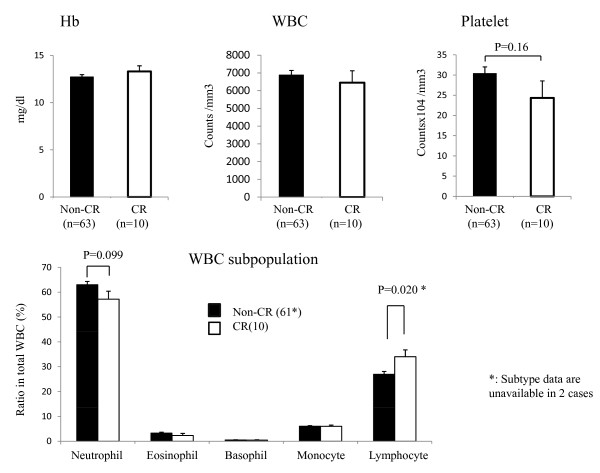
**Hemoglobin (Hb), white blood cell (WBC) and platelet counts (A) as well as white blood cell subpopulations (B) in circulating blood taken before CRT in 10 CR and 63 non-CR cases**. *: p < 0.05 by paired t-test.

In the 73 patients, blood data on hemoglobin (Hb), white blood cells (WBC) with their subpopulations, and platelets were examined at various time points before CRT and during and after CRT until surgery. First, we evaluated blood cell data before CRT in 10 CR and 63 non-CR cases. None of Hb, WBC and platelet counts showed any significant difference between CR and non-CR cases. Interestingly, however, the numbers of lymphocytes and neutrophils showed different associations with tumor response. As shown in Figure 1, CR cases showed a relatively lower neutrophil count, while lymphocyte count tended to be higher in CR cases. If the percentage of lymphocytes in the total WBC population was compared, CR cases had a significantly higher percentage of lymphocytes than that in non-CR cases (p = 0.020). With multivariate stepwise logistic regression analysis, the pre-CRT lymphocyte percentage, but not tumor size, showed an independent correlation with CR rate (Table 2).

**Table 2:** Multivariate analysis of Complete response (CR) rate

Variable	Odds (95% CI)	p value	
Circumferential extent (>60% vs. ≤60%)	3.833	0.115	
Platelet count	1.063 (0.983-1.178)	0.141	
% Lymphocytes in WBC	0.676 (0.415-0.947)	0.019	
% Neutrophils in WBC	0.735 (0.478-1.004)	0.054	

The independence of five factors with a possible correlation with CR rate were analyzed by stepwise logistic regression analysis using JMP software 8.0.

**Figure 2:**
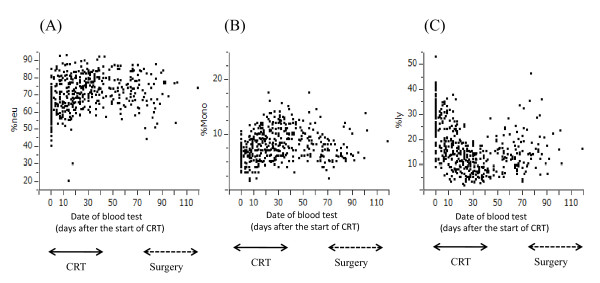
**Change in ratio of neutrophils (A), monocytes (B) and lymphocytes (C) in peripheral blood samples during entire preoperative treatment period. Each dot shows the values obtained from all the patients during this period**. As compared with neutrophils and monocytes, the lymphocyte percentage was markedly reduced during CRT and gradually recovered over several weeks after the end of CRT until surgery.

**Figure 3:**
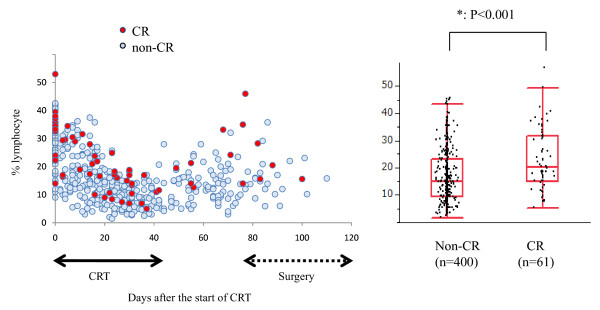
**Ratio of lymphocytes in WBC tended to be maintained at a relatively higher level in 10 CR cases as compared with 63 non-CR cases**. When all the blood data were grossly compared, statistical significance was obtained. *: p < 0.001 by ANOVA.

However, the blood cell counts appeared to change during the treatment period. Therefore, we next examined the numbers of leukocyte subpopulations, i.e., neutrophils, monocytes and lymphocytes, in all the blood samples taken from these patients from the start of CRT to surgery (or to the first biopsy in 3 CR patients who did not undergo surgery). As shown in Figure 2, the numbers of circulating neutrophils and monocytes were relatively stable during the treatment period. In contrast, the number of circulating lymphocytes was markedly reduced during CRT and showed a gradual increase up to the time of surgery. When the lymphocyte count in the total blood samples was compared between CR and non-CR cases, samples derived from the CR group tended to contain more lymphocytes than those from the non-CR group (Figure 3). In contrast, neutrophil percentage was higher in non-CR cases (data not shown). Because this was a retrospective study and the timing and frequency of blood tests varied markedly among patients, the comparison may not be significant from the statistical point of view, and a prospective study is necessary to draw a firm conclusion on this point. However, our data raise the possibility that circulating lymphocytes may have significant biological effects on the tumor response to CRT.

Peripheral lymphopenia, especially reduced T lymphocytes, after RT was first described in the 1970's [11,12], but the clinical significance of this drop in the circulating lymphocyte count has not been well evaluated. A literature search yielded no previous report of a significant correlation between circulating lymphocyte count and RT response. However, the degree of recovery of lymphocyte count after RT has been shown to correlate with tumor recurrence [13,14]. These facts allow us to speculate that the radiation-induced depression of circulating lymphocyte may provide an opportunity for re-growth via proliferation of tumor cells that survived the irradiation damage, thereby reducing the likelihood of CR after RT.

In fact, radiosensitivity has been shown to be dependent not only on the biological characteristics of tumor cells but also on the tumor microenvironment [15,16]. Although circulating leukocyte count reflects the host immune status, neutrophils usually act as the first responders to microbial infection in acute inflammation, while lymphocytes recognize specific "non-self" antigens and eliminate a specific pathogen or pathogen-infected cells. Since tumor cells usually have a tumor-associated antigen, lymphocytes, especially T cells, are thought to play a central role in anti-tumor immunity, and the absolute number of host lymphocytes could be biologically relevant for tumor response to CRT. Since the first report in 1979, [17], it has been proposed that tumor shrinkage is not simply dependent on direct damage to irradiated tumor cells, but also to be greatly affected by the host immune response [18]. In fact, in vivo studies have suggested that cancer cells, dead or dying due to radiotherapy or chemotherapy, can present tumor-associated antigens to host immune cells and thereby evoke anti-tumor immune responses [19,20]. Moreover, accumulating clinical data suggest the presence of radiation-induced anti-tumor immunity in humans [21,22]. Therefore, the marked reduction in the circulating lymphocyte count during CRT may be a significant disadvantage for patients. Together with these facts, our observations suggest the possibility that the lymphocyte-mediated immune response against damaged tumor cells is important for achieving CR during CRT in RC cases.

In our data, the association between lymphocyte ratio and clinical efficacy was observed in primary tumor, but not in metastatic lymph nodes (data not shown). Since tumor shrinkage is more dependent on local immune response, this may be reasonable that the clinical effects during CRT are largely different between in lymph nodes and in primary tumors. Further analysis on tumor infiltrating lymphocytes (TIL) in malignant tissues is essential to see the accurate contribution of host immune reaction on CRT response.

In contrast to lymphocytes, the neutrophil count showed an inverse correlation with tumor response. An increase in neutrophil count usually reflects an acute inflammatory response against bacterial infection. In our series, other inflammatory markers, such as platelet count and serum levels of C reactive protein (CRP) and fibrinogen also showed a similar association, although not statistically significant (data not shown). Previous studies have shown that neutrophils can suppress the T cell response through the production of reactive oxygen species (ROS), nitric oxide (NO) and arginase [23,24]. This suggests that the presence of an acute inflammatory response during CRT may cause suppression of lymphocyte-mediated immunity through increased circulating neutrophils and thus elicit unfavorable effects on tumor response.

Although the results obtained from this retrospective analysis have limitations, the significant association between the circulating lymphocyte number and CR rate supports the hypothesis that total eradication of tumor cells after CRT is dependent, at least in part, on host immune reaction. Enhancing lymphocyte-mediated immunity during CRT may be a lead to the improvement of the clinical efficacy of CRT in RC patients. Further analysis of the phenotypic and functional characteristics of circulating as well as tumor infiltrating lymphocytes may clarify the novel mechanisms underlying the responsiveness of tumors to CRT.

## Competing interests

The authors declare that they have no competing interests.

## Authors' information

JK participated in the study design and data retrieval and analysis. KY, KK, ES participated in data retrieval and analysis. HN participated in the management of this study. All authors read and approved the final manuscript.
